# Towards a health promoting university: descriptive findings on health, wellbeing and academic performance amongst university students in Australia

**DOI:** 10.1186/s12889-022-14690-9

**Published:** 2022-12-27

**Authors:** Lena Sanci, Ian Williams, Melissa Russell, Patty Chondros, Ann-Maree Duncan, Laura Tarzia, Divya Peter, Madeleine S. Y. Lim, Adrian Tomyn, Harry Minas

**Affiliations:** 1 Department of General Practice, Melbourne Medical School, The University of Melbourne, Melbourne, Australia; 2 Centre of Epidemiology and Biostatistics, Melbourne School of Population and Global Health, The University of Melbourne, Melbourne, Australia; 3 School of Psychology, Deakin University, Melbourne, Australia; 4 Melbourne School of Population and Global Health, The University of Melbourne, Melbourne, Australia

**Keywords:** University student, Young person, International student, Mental health, Academic outcomes

## Abstract

**Background:**

Universities are increasingly recognised as institutions where health and wellbeing can be promoted to maximise academic outcomes, career transitions, and lifelong positive health behaviours. There is concern about the mental health of university students and other factors which affect academic outcomes particularly for subgroups such as international students. There are few cohort studies of the breadth of issues that can impact on mental health and academic outcomes for both local and international students. We conducted a baseline prevalence survey of students at a large Australian university covering health, academic, and social determinants of wellbeing. The purpose was to inform the university’s new student health and wellbeing framework with a view to follow-up to determine predictors of mental ill-health and academic outcomes in the subsequent year. In this paper we present the baseline prevalence data and report on selected mental health and health care access issues for local and international students.

**Methods:**

The entire university population as of April 2019 of over 56,375 students aged 18 or above were invited to complete the online survey. Questions explored eight domains: demographic characteristics, general health and wellbeing, mental health, risk taking behaviours, psychosocial stressors, learning and academic factors, social and cultural environment, and awareness of and access to health and wellbeing services. Records of academic results were also accessed and matched with survey data for a large subset of students providing consent.

**Results:**

Fourteen thousand eight hundred eighty (26.4%) students commenced our survey and were representative of the entire student population on demographic characteristics. Three quarters were aged between 18 to 25 years and one third were international students. Eighty-five percent consented to access of their academic records. Similar proportions of local and international students experienced symptoms of a depression or anxiety disorder, however international students were less aware of and less likely to access available health services both inside and external to the university. We also reported on the prevalence of: general lifestyle factors (diet, exercise, amount of daily sleep); risk-taking behaviours (including alcohol, tobacco and other drug use; unprotected sexual activity); psychosocial stressors (financial, intimate partner violence, discrimination, academic stressors, acculturative stress); subjects failed; resilience; social supports; social media use; and health services accessed online.

**Conclusions:**

This rigorous and comprehensive examination of the health status of local and international students in an Australian university student population establishes the prevalence of mental health issues and other psychosocial determinants of health and wellbeing, along with academic performance. This study will inform a university-wide student wellbeing framework to guide health and wellbeing promotion and is a baseline for a 12-month follow-up of the cohort in 2020 during the COVID-19 pandemic.

**Supplementary Information:**

The online version contains supplementary material available at 10.1186/s12889-022-14690-9.

## Background

Universities offer profound opportunity to positively impact young people’s healthy development. They host large numbers of young people as they progress into early adulthood and juggle an expanding array of opportunities and life experiences. University enrolments have been increasing in Australia and internationally [1–6] and, at least prior to COVID-19, international student numbers were growing with universities as centres of global learning [4, 5]. As well as being academic institutions, universities are integrated settings suited to promoting healthy development, learning, and social life. They are also well-positioned to develop, test and deliver best practice interventions, given their strong academic and research skillsets [7]. Furthermore, post-secondary students in high, middle, and low-income countries are regarded as a key population for influencing economic growth and success, not only of the students themselves, but of the country as a whole [6].

The university sector is increasingly aware of the need to support and promote the health and wellbeing of students to optimise opportunities for academic success and readiness to transition to the workforce [3, 7–9]. Young adults entering university have been reported to experience mental disorders and severe psychological distress at rates even higher than age-matched peers in the general population [10, 11]. Depression in university students has been linked to lower academic performance [12], interpersonal relationship issues [13], suicide risk [14] and workplace performance issues [15]. Additional challenges for university students include difficulty balancing academic workloads with other responsibilities [16], performance pressure [17], homesickness [18], financial pressures, and worry about future debt [19–21]. A recent World Health Organisation (WHO) study of first year students from eight countries assessed the magnitude and nature of student emotional problems, along with their impact on the student experience, academic outcomes and barriers to seeking treatment [22]. Initial results have been published on mental disorder prevalence, with around one third of fulltime students screening positive for common mental disorders (e.g. major depression, generalised anxiety, alcohol and substance use disorders) [23].

International students frequently experience additional factors that may negatively impact on their mental health and educational outcomes such as culture shock, parental and cultural pressure to succeed, social isolation, language proficiency issues, discrimination, and financial pressures [24, 25]. Visa insecurity can also render them more vulnerable to interpersonal violence [26]. International students may also be less likely to seek help for these concerns, especially for mental health issues [27, 28]. Recent studies have reported these pressures exist for international students studying in Australia [29–32].

Students’ vulnerability to mental health concerns is perhaps not surprising given that transition to university occurs within the pathway of development from adolescence to young adulthood, a period during which risks for current and future health compromise emerge [33]. Patterns of substance use, obesity and low rates of physical exercise lay the foundations for non-communicable diseases later in life [34, 35]. Half of all adult mental disorders begin by the age of 14 years and 75% by 24 years [36]; injuries and reproductive health risks are common in young adults [33–35]. Furthermore, young people aged 16–24 are the highest risk group for sexual violence victimization [37], and around one in five young Australian adults attending primary care for routine visits report having experienced fear or abuse in intimate partner or family relationships [38].

Research examining the health and wellbeing of university students and their awareness and use of existing services, particularly in Australia, is emerging [29, 32, 39]. To date however, no large-scale studies have examined the prevalence of these factors, in conjunction with academic performance, in a representative sample of university students. The current study addresses this evidence gap through a cross-sectional examination of a broad range of health and wellbeing factors affecting the mental health and academic performance of an Australian university student population, using a university-wide anonymous survey. The goals of this initial survey were to form the baseline for a follow-up study of the cohort and to inform potential strategies for universities to move toward becoming mental health and wellbeing promoting institutions. In particular, this study examined the experiences of both local and international students to identify how the tertiary education system may need to respond with both universal and specific strategies for each group. The follow-up study has occurred during the COVID-19 pandemic, hence, impacts on mental health from changes such as online learning and international students either remaining in Australia or remaining or returning to their home country, can be assessed. Predictors of vulnerability during COVID-19 can be examined using the baseline work reported in this paper to shed further light on how universities might tailor support strategies in times of crisis.

In this paper we describe the study design and present descriptive findings on key health and wellbeing issues reported by participating local and international students across the domains of:

- general health and wellbeing, mental health, risk taking behaviours, and psychosocial stressors;
- learning and academic factors;
- social and cultural environment; and
- awareness of, and access to, health and wellbeing services.

Anticipated future papers will provide greater detail on each domain and expand on the patterns of wellbeing and risk for subgroups and examine the effect of factors such as social media use, risk taking behaviours, physical factors, experience of violence and being afraid of an intimate partner on mental health and academic performance.

## Methods

### Study design and setting

This study was undertaken at a large, established, and high-ranking metropolitan Australian university in the state of Victoria between 2^nd^ April and 3^rd^ June 2019. During each study phase, from project commencement to completion, the project team was advised by a stakeholder advisory group comprising representatives from student associations, University Chancellery Departments, Residential Colleges, the philanthropic arm of the funding body, and other university researchers with experience in university student wellbeing.

The study was approved by the research institution’s Human Research Ethics Committee (Ethics ID 1,852,199).

### Participants

#### Recruitment, study size and bias

All 56,375 students currently enrolled at the university in March 2019 and aged 18 and above based on the University records were invited to complete an online survey. Students enrolled in non-award courses, cross-institutional or exchange programs, and those whose status was ‘not admitted’ (e.g. due to leave from study and potential course completion) were ineligible.

A diverse range of recruitment and promotion activities were instigated across the university two weeks prior to survey launch including: printed posters, flyers, and faculty newsletters; digital slides for academic lecturers in each faculty; postings to online student social media channels and the university’s main student web portal; and a short promotional video made by students explaining the objectives and methods of the study. Students were offered an incentive to participate of entry to a random prize draw for the chance of winning one of over 50 prizes (including iPads, cycle vouchers, and gift cards) upon completion of the survey.

Participation in the survey was voluntary and under conditions of informed consent. All eligible participants were emailed an invitation to complete the survey via a unique URL; responses were tracked using an anonymous study identity number (ID). Reminder emails were sent to non-responders on a weekly basis during the eight weeks that the survey was open (pattern of surveys completed following each reminder are shown in Supplementary Fig. 1). At the end of the survey, and at key sections asking about sensitive issues, information about crisis and support services was provided to address potential distress or concerns.

### Survey design

The survey covered a wide range of variables relevant to mental health, wellbeing, and academic outcomes, distilled into eight broad domains: demographic characteristics; general health and wellbeing; mental health; risk taking behaviours; psychosocial stressors; learning and academic factors; social and cultural environment; and awareness of and access to health and wellbeing services. Students were also asked for their consent to link their survey results to their academic performance transcripts. Students not consenting were asked to self-report any failed subjects in their course of study. Comprising over 130 items drawn from validated scales and purpose-designed items, the survey included skip logic and various branching questions to minimise survey length for each participant.

The online survey was distributed and managed using the Qualtrics XM survey platform (Qualtrics, Provo, UT). It was pilot tested in a four-hour workshop with a group of 15 students recruited from our student stakeholders’ networks. Students provided feedback on framing and comprehension of questions, survey length and item order. The survey was refined and shortened in response, leading to a final questionnaire that could be completed in approximately 20 min. Students also had input on marketing, recruitment, incentive materials and strategies, and acceptability of linkage to academic transcripts.

#### Measures

Measures employed in the student survey (see Table 1) were organised across eight survey domains (see Supplementary Fig. 2). Derivation of scores for scales is described in Supplementary Appendix 1. A selection of these measures is presented in this paper. Measures were identified following a literature search using a combination of key search terms (including synonyms for *university student*, *health risk factor*, *academic achievement*, *health service access*, and *student risk factor*). Stakeholder input was sought before the final list of measures for each domain were selected.

**Table 1:** Summary of scales and measures^a^ used in student survey

Domain	Scale/item Content	No. Items
**Demographics**		
-various	age, gender, residential status, country of birth, age of arrival in Australia, Aboriginal or Torres Strait Islander heritage, English language competency, highest academic qualification, current living arrangements, hours of paid work; enrolment characteristics (incl. faculty, level of study, area of study, current enrolment type, academic year, attendance mode, hours spent on campus, course fee paying status), private health insurance, relationship status	20
**General Health and Wellbeing**		
-self-reported general health	from Short Form Health Survey (SF-12) [40]	1
-height & weight	self-report height and weight [41]	2
-nutrition	daily serves of fruit and vegetables [41]	2
-sleep	average sleep per night^b^	1
-physical activity	weekly physical activity [41]^(adapted)^	1
-chronic health condition	chronic physical condition or disability^b^	1
-sexual & reproductive health	sexual orientation^b^	1
	sex education [42]^(adapted)^	1
	meeting sexual partner/s^b^	1
	contraceptive use [42]^(adapted)^	2
**Mental Health**		
-self-reported mental health	self-reported mental health condition [43] concern about current mental/emotional state [44]	1 1
-depressive symptoms^c^	Patient Health Questionnaire-9 (PHQ9) [45]	9
-anxiety symptoms^c^	Generalized Anxiety Disorder-7 [46]	7
-suicidality	self-harm or suicide attempts [47]^(adapted)^	1
-eating disorder symptoms^c^	Sick Control One stone Fat Food (SCOFF) [48]	5
-resilience^c^	Connor–Davidson Resilience Scale (CD-RISC) [49]	10
**Risk Taking Behaviours**		
-tobacco use	self-reported tobacco use [43, 50]^(adapted)^	3
-hazardous alcohol use^c^	Alcohol Use Disorders Identification Test (AUDIT-C) [51]	3
-illicit drug use	self-reported illicit drug use [43, 50]^(adapted)^	3
-sex	past sexually transmitted infection (STI) [42]	1
	number of sexual partners [42]^(adapted)^	1
	sexual partners overlapping in time^b^	1
	experiences of unwanted sex [42]^(adapted)^	1
-perpetration of intimate partner violence	perpetration of controlling, threatening, physically abusive, fear-inducing or sexually abusive behaviour^b^	5
**Psychosocial Stressors**		
-financial concerns	difficulty affording food or medication [52]^(adapted)^	2
	providing services in return for accommodation^b^	1
	homelessness^b^	1
-unwanted sexual contact	experiences of forced/unwanted sexual contact [53, 54]	6
-intimate partner fear/violence^c^	Self-reported fear of partner [55]	30
	Composite Abuse Scale (CAS) [56]	
-acculturative stress^c^	Social, Attitudinal, Familial and Environmental Acculturative Stress Scale (SAFE) [57]	13
-discrimination	experiences of discrimination at university and/or in the wider community [58]^(adapted)^	2
-academic stressors	a range of possible academic stressors experienced at university [29]^(adapted)^	1
**Learning and Academic Factors**		
-semester subject failures^d^	number of subjects failed in Semester 1, 2019	N/A
-average grades^e^	self-report of average overall grade during current course of study^b^	1
-course subjects failed^e^	self-report of any subjects failed during current course of study^b^	1
-considered dropping out	considered dropping out from current course and reasons why^b^	2
-learning style^c^	Adelaide Diagnostic Learning Instrument, Brief (ADLIB) [59]	21
**Social and Cultural Environment**		
-social support^c^	Medical Outcomes Study Social Support Survey (MOS-SSS) [60]	6
-friendship groups	friendship groups in Australia^b^	1
-organised social/sport group	involvement in organised social, sporting or recreational groups [50]^(adapted)^	1
-cultural values	values relating to family, individualism, achievement, hedonism and reserve^b^	10
-intimate relationships	relationship status [41]^(adapted)^	1
-social media use	social media use and related stress^b^	2
-health service awareness	awareness of university student health/wellbeing and support services [44]^(adapted)^	1
-health service access	use of university student support services and university or community health/wellbeing services^b^; [41]; [44]^(adapted)^	4
-health information & online services	sources of health information consulted [61]^(adapted)^	1
	use of online health and wellbeing services [62]^(adapted)^	1
-unmet need	unable to access mental health care or general health care when it was needed [50, 61]^(adapted)^	2
-barriers to service use	barriers experienced in accessing mental or general health care [50, 61]^(adapted)^	2

^a^Not all items were presented to every participant (see text for details); not all measures are reported in the present paper; derivation of scores from scales is described in Supplementary Appendix 1

^b^Item/s devised by research team

^c^Derivation of scale scores explained in Supplementary Appendix 1

^d^Data obtained from university academic records from students consenting to linkage with academic records

^e^Measured only in students not consenting to linkage with academic records, but not reported in this paper

Wherever possible we employed scales or measures that had previously been validated [45, 46, 48, 56] and used in studies with young adult populations [21, 29, 63–66]. Measures previously used in studies of tertiary students were prioritised, particularly those in Australian settings where the cultural and international student profile is similar to the present study [29, 44, 47, 52, 58]. Other measures were derived from studies of Australian young people [42, 43, 50, 62, 67], or from Australian population surveys or studies that encompass this age group [60, 68]. We also consulted grey literature and technical reports of university-based surveys [69, 70]. Several additional questions were developed or modified by the study authors where no suitable measures were identified in the literature (e.g. items on social media induced stress and university health service access).

We defined a local student to be an Australian or overseas-born student who is an Australian citizen or permanent resident; international students were defined as those holding an Australian temporary resident student visa or bridging visa and who have come to Australia to study.

### Statistical analysis

Descriptive statistics are used to summarise participant characteristics overall and by international and local student citizenship status. Means and standard deviation (SD) are used for continuous variables (such as age in years). Categorical variables are summarised as counts, and the denominators for all sample percentages are based on those participants who provided a response to the relevant scale or survey item.

To adjust for potential response bias in the sample percentages we provided weighted percentages calculated using inverse probability weights (IPW) using the STATA 17 [71] *survey* command. We first predicted each student’s probability weight of response by fitting a logistic regression on all the students who were sent an invitation to complete the survey. The dependant variable (outcome) was a binary response of whether the student responded to the survey (1) or not (0) and the predictor variables were gender (male/female/self-described) and citizenship (local and international students). Further detail about this analysis is in Supplementary Appendix 2.

The summary statistics by responders and non-responders and the response probabilities were calculated in SPSS [72]. All other analyses were conducted using STATA 17 Statistical software [71].

## Results

### Population profile

**Fig. 1:**
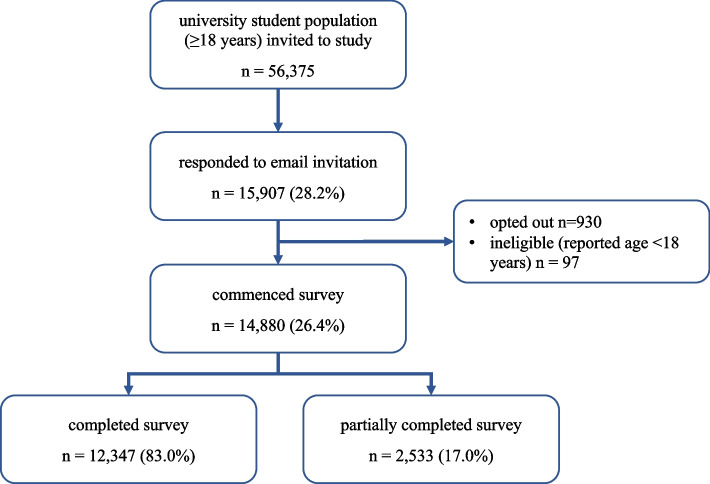
Flowchart showing participant recruitment and survey completion

From a total of 56,375 students who were 18 years and older according to university records and were invited to participate in the survey, 15,907 (28%) clicked the invitation link, and  930 subsequently opted out (38 did not consent to the survey and 892 students consented but exited the survey before completing the survey questions). Of the students who responded and were aged 18 and over according to the university records, 97 students self-reported age being under 18 years and thus were excluded from the analysis (see Fig. 1). Our final sample comprised 14,880 students, representing over one quarter (26.4%) of the initial student pool.

### Survey sample

The majority of the 14,880 students (*n* = 12,347, 83%) responded to all sections of the survey. The students who did not complete the survey (*n* = 2,533, 17%) mostly exited within the initial sections (Supplementary Table 1 shows the response rate by survey section). The question asking students for consent to linking their survey answers with their university academic records was at the end of the first section, and 85% (*n* = 12,225) of the 14,390 who answered this consent question agreed to this linkage. Respondents’ self-reported characteristics (age, gender, broad course level, and Faculty) closely matched the whole university’s student population profile (see Supplementary Table 2). In this section, we describe the self-reported characteristics of the survey respondents (sample).

#### Sample demographic characteristics

Table 2 shows the demographic characteristics of the sample. Just under two thirds of the 14,880 survey respondents reported that they were local students, which was representative of the population (Supplementary Table 2). The mean age of the local and international students was similar with almost three quarters (74.1%, *n* = 11,027) aged between 18 and 25 years. Just over one quarter of local students (27.2%, *n* = 2558), were not born in Australia, with a mean age of arrival in Australia of 13.4 (SD = 9.4) years (result not shown in Table 2). Of local students born in Australia, 1.4% (*n* = 95) identified as Aboriginal or Torres Strait Islander (result not shown in Table 2). Almost all international students were born overseas (*n* = 5474) with a mean age of arrival in Australia at 21.8 (SD = 4.9) years (result not shown in Table 2). Almost half of international students (48.7%, *n* = 2638) were born in China, with smaller proportions originating in countries such as India, Malaysia, Indonesia, Singapore, Hong Kong, Vietnam, Europe, and United States (result not shown in Table 2). Most (96.4%, *n* = 5178) international students were in full-time study compared to 85.5% (*n* = 12,503) of local students (Supplementary Table 2). Almost two thirds of international students were enrolled in postgraduate studies compared to about half of local students (Table 2).

**Table 2:** Demographics of sample (*N* = 14,880) by Local (*N* = 9,398) and International students (*N* = 5,482)

	**Total**		**Local**		**International**	
	**Mean**	**SD**	**Mean**	**SD**	**Mean**	**SD**
**Age (years)**	24.19	6.88	24.46	7.95	23.71	4.44
	**n**	**%**	**n**	**%**	**n**	**%**
**Gender**						
Male	5251	35.6	3282	35.1	1969	36.3
Female	9432	63.9	5987	64.1	3445	63.6
Self-describe	77	0.5	71	0.8	6	0.1
**Faculty**						
Architecture, Building and Planning	818	5.6	391	4.2	427	8.0
Arts	2480	17.0	1720	18.6	760	14.2
Business and Economics	2095	14.3	973	10.5	1122	20.9
Education	800	5.5	618	6.7	182	3.4
Engineering	1275	8.7	373	4.0	902	16.8
Fine Arts and Music	521	3.6	471	5.1	50	0.9
Law	545	3.7	398	4.3	147	2.7
Medicine, Dentistry and Health Sciences	2546	17.4	1968	21.3	578	10.8
Science	2884	19.7	1935	20.9	949	17.7
Veterinary and Agricultural Sciences	552	3.8	322	3.5	230	4.3
Other	109	0.7	85	0.9	24	0.4
**Course level**						
Undergraduate	6830	46.7	4804	51.9	2026	37.7
Postgraduate Coursework	6001	41.0	3352	36.2	2649	49.3
Other postgraduate	346	2.4	278	3.0	68	1.3
Research Higher Degree	1448	9.9	820	8.9	628	11.7
**Highest qualification completed**						
High School or equivalent	6373	43.2	4484	48.1	1889	34.9
Vocational program or associate degree	142	1.0	108	1.2	34	0.6
Bachelor Degree	6036	40.9	3464	37.2	2572	47.5
Postgraduate studies	2190	14.9	1268	13.6	922	17.0
**Provided consent to access official academic records**^a^	12225	85.0	7885	86.2	4370	82.8

Counts (n) and percentages (%) presented, unless otherwise stated; *SD* Standard deviation

^a^Total number of students who were asked for consent = 14,390; 9,115 Local students and 5,275 International students (490 students had dropped out of the survey by the stage at which this question was asked – Refer to Supplementary Table 1)

#### General health and wellbeing

While most students (80.3%) gave positive ratings of their health (*good*, *very good* or *excellent*), a majority failed to meet health recommendations for physical activity (30 min or more of moderate intensity physical activity on five or more days per week [73]) and nutrition (two daily serves of fruit [68] and five to six daily serves of vegetables [74]) (see Table 3). Based on Body Mass Index (BMI) calculated from self-reported height and weight, over two thirds of students were in the healthy weight range (BMI: 18.5 to 24.9) [75]. Of those in the unhealthy weight range, a greater proportion of local students were in the overweight/obese categories while more international students were in the underweight range (Table 3). Most students reported their average nightly sleep as falling within the recommended levels for young adults (seven to nine hours per night [76]).

**Table 3:** General Health and Wellbeing all students (*N* = 14,880) and by Local (*N* = 9,398) and International students (*N* = 5,482)

	**Total**			**Local**			**International**		
	**n**	**%**	**Wt %**	**n**	**%**	**Wt %**	**n**	**%**	**Wt %**
**In general, would you say your health is…**									
Excellent	1640	12.6	13.1	1206	14.5	15.1	434	9.3	9.7
Very Good	4290	33.0	33.2	2804	33.7	33.9	1486	31.7	31.9
Good	4456	34.3	34.0	2548	30.7	30.4	1908	40.7	40.3
Fair	2122	16.3	16.1	1372	16.5	16.2	750	16.0	15.8
Poor	496	3.8	3.7	383	4.6	4.5	113	2.4	2.3
**Physical activity at least 30 min**									
0–2 days/week	5601	43.1	42.5	3223	38.8	38.2	2378	50.7	49.8
3–4 days/week	4383	33.7	33.8	2951	35.5	35.5	1432	30.5	31.0
5 or more days/week	3020	23.2	23.7	2139	25.7	26.3	881	18.8	19.2
**Serves of fruit/day**									
0–1 serves	6470	51.3	51.7	3935	48.1	48.4	2535	57.2	57.6
2 or more serves	6135	48.7	48.3	4242	51.9	51.6	1893	42.8	42.4
**Serves of vegetables/day**									
0–2 serves	6370	51.4	51.8	3545	43.7	44.1	2825	66.0	66.0
3–4 serves	4675	37.7	37.4	3502	43.1	42.8	1173	27.4	27.4
5 or more serves	1354	10.9	10.8	1069	13.2	13.1	285	6.7	6.6
**Body Mass Index **[75]									
Underweight (under 18.5)	1186	9.2	8.7	588	7.1	6.8	598	12.8	11.9
Healthy weight (18.5 to 24.9)	8898	68.8	68.6	5729	69.3	69.2	3169	67.8	67.5
Overweight (25.0 to 29.9)	2129	16.5	17.1	1426	17.3	17.7	703	15.1	16.2
Obese (over 30)	722	5.6	5.6	521	6.3	6.3	201	4.3	4.5
**Average hours of sleep/night**									
5 h or less	817	6.3	6.2	484	5.8	5.8	333	7.1	7.0
6 h	2688	20.7	20.7	1678	20.2	20.2	1010	21.5	21.4
7 h	5048	38.8	39.0	3286	39.5	39.7	1762	37.6	37.8
8 h	3569	27.4	27.4	2287	27.5	27.5	1282	27.3	27.3
9 h or more	882	6.8	6.7	578	7.0	6.9	304	6.5	6.4

Discrepancies in totals due to missing responses

#### Mental health

Overall, students reported good mental health, with the majority showing no or low signs of depression, anxiety or eating disorder, and moderate levels of resilience (scoring a mean of 26 out of 40 where a higher score equates with more resilience) (Table 4). Nevertheless, about one in five of both local and international students reported ‘a lot’ or ‘a great deal’ of concern about their current mental/emotional state and 26.5% of local students and 14.0% of international students reported currently having a mental health condition. Moderate to severe depressive symptoms in the preceding two-week period as measured by the PHQ-9 were experienced by 30.7% of local students and 25.9% of international students. Fewer students (24.8% of local and 19.8% of international students) reported moderate to severe anxiety symptoms in the previous 2 weeks as measured by the GAD-7. About one in five of both local and international students endorsed items suggesting the possibility of an eating disorder (SCOFF screening questionnaire). Almost five percent of all students reported self-harm or suicide attempts in the previous 12 months.

**Table 4:** Mental Health for all students (*N* = 14,880) and by Local (*N* = 9,398) and International students (*N* = 5,482)

	**Total**			**Local**			**International**		
	**Mean**	**SD**	**Wt Mean**	**Mean**	**SD**	**Wt Mean**	**Mean**	**SD**	**Wt Mean**
**Resilience** (CD-RISK)^a^ [49]	26.40	7.07	26.53	27.18	6.93	27.32	25.01	7.09	25.15
	**n**	**%**	**Wt %**	**n**	**%**	**Wt %**	**n**	**%**	**Wt %**
**Self-reported current mental health condition**									
Yes	2965	22.8	21.9	2292	27.6	26.5	673	14.4	14.0
No	10,016	77.2	78.1	6014	72.4	73.5	4002	85.6	86.0
**Concerned for current mental health or emotional state**									
Not at all	2640	20.3	21.0	1638	19.7	20.3	1002	21.4	22.0
A little	3988	30.7	30.8	2563	30.9	30.9	1425	30.5	30.6
Somewhat	3528	27.2	26.9	2181	26.3	26.0	1347	28.8	28.4
A lot	1924	14.8	14.6	1263	15.2	14.9	661	14.1	13.9
A great deal	901	6.9	6.8	661	8.0	7.8	240	5.1	5.0
**Depressive symptom** (PHQ-9)^a^ [45]									
Minimal (0–4)	4559	35.4	36.2	3004	36.4	37.3	1555	33.6	34.3
Mild (5–9)	4492	34.9	34.8	2644	32.1	31.9	1848	40.0	39.8
Moderate (10–14)	2172	16.9	16.5	1431	17.4	17.0	741	16.0	15.7
Moderately severe (15–19)	1041	8.1	7.9	724	8.8	8.5	317	6.9	6.8
Severe (20–27)	607	4.7	4.6	444	5.4	5.2	163	3.5	3.4
**Anxiety symptom** (GAD-7)^a^ [46]									
Minimal (0–4)	5593	43.8	44.9	3489	42.5	43.8	2104	45.9	46.8
Mild (5–9)	4161	32.6	32.1	2604	31.8	31.4	1557	34.0	33.4
Moderate (10–14)	1812	14.2	13.9	1213	14.8	14.4	599	13.1	12.9
Severe (15–21)	1216	9.5	9.1	894	10.9	10.4	322	7.0	6.9
**Probable anorexia or bulimia** (SCOFF)^a^ [48]									
Yes	2836	22.3	21.2	1756	21.5	20.4	1080	23.7	22.7
No	9897	77.7	78.8	6419	78.5	79.6	3478	76.3	77.3
**Tried to harm or kill yourself in past 12 months**									
Yes	607	4.9	4.8	434	5.5	5.2	173	3.9	3.9
No	11699	95.1	95.2	7467	94.5	94.8	4232	96.1	96.1

Counts (n) and percentages (%) presented, unless otherwise stated; *SD* Standard deviation

Discrepancies in totals due to missing responses

^a^Total students without a missing score = 12,460; 8,004 Local students and 4,456 International students

#### Risk taking behaviours

Local students reported more risk-taking behaviours than international students (Table 5). About two in three local students were drinking at hazardous levels (based on frequency and volume of consumption) in the past year compared to about one in three international students. Over one in ten students (13.9% of local and 10.9% of international) reported having smoked tobacco in the past 12 months. Of those who smoked, one third of international students reported smoking every day compared to one in five local students. About one in four local students and one in sixteen international students reported using illicit drugs (such as marijuana, heroin, cocaine, or ecstasy) in the past 12 months.

**Table 5:** Risk Taking Behaviours for all students (*N* = 14,880) and by Local (*N* = 9,398) and International students (*N* = 5,482)

	**Total**			**Local**			**International**		
	**n**	**%**	**Wt %**	**n**	**%**	**Wt %**	**n**	**%**	**Wt %**
**Potentially hazardous drinking in past 12 months** (AUDIT-C) [51]									
Did not drink	2270	18.2	18.2	1091	13.5	13.6	1179	26.7	26.4
Drinks, but not hazardous	3823	30.6	30.1	1959	24.2	23.7	1864	42.2	41.4
Hazardous drinking	6407	51.3	51.7	5038	62.3	62.7	1369	31.0	32.1
**Smoked cigarette in past 12 months**									
Yes	1580	12.4	12.8	1111	13.6	13.9	469	10.3	10.9
No	11167	87.6	87.2	7074	86.4	86.1	4093	89.7	89.1
**Frequency smoked cigarettes in past 12 months**^a^									
once a month/weekends	735	48.1	47.5	585	54.2	54.0	150	33.4	32.8
Once/twice during week	195	12.8	12.7	126	11.7	11.7	69	15.4	15.2
3 + times a week, not daily	230	15.0	15.0	151	14.0	13.9	79	17.6	17.4
Everyday	369	24.1	24.7	218	20.2	20.4	151	33.6	34.7
**Used drugs (marijuana and/or other) in past 12 months**									
Yes	2341	18.9	19.1	2074	26.1	26.4	267	6.0	6.2
No	10058	81.1	80.9	5871	73.9	73.6	4187	94.0	93.8
**Experienced intercourse (vaginal and/or oral) in past 12 months**									
Yes	6387	58.9	59.1	4644	65.1	65.2	1743	46.9	47.6
No	4461	41.1	40.9	2486	34.9	34.8	1975	53.1	52.4
**Used condoms when having sex in past 12 months**									
Always	2350	37.4	37.9	1425	31.2	31.5	925	54.0	54.3
Sometimes	2191	34.9	35.0	1701	37.2	37.4	490	28.6	28.8
Never	1743	27.7	27.1	1446	31.6	31.0	297	17.3	16.9
**Ever been diagnosed with sexually transmitted infection**									
Yes	628	5.0	5.0	523	6.5	6.4	105	2.4	2.4
No	11842	95.0	95.0	7541	93.5	93.6	4301	97.6	97.6

^a^For students who smoked cigarettes in past 12 months (Total students with a response = 1529; 1,080 Local students and 449 International students)

Overall, 6.4% of local and 2.4% of international students reported having ever been diagnosed with a sexually transmissible infection (STI) (Table 5). About one third of the local students and just over half of the international students reported always using a condom when they had sex.

#### Psychosocial stressors

Students were asked about a wide range of possible stressors they may encounter during university life (Table 6).

**Table 6:** Psychosocial Stressors for all students (*N* = 14,880) and by Local (*N* = 9,398) and International students (*N* = 5,482)

	**Total**			**Local**			**International**		
	**n**	**%**	**Wt %**	**n**	**%**	**Wt %**	**n**	**%**	**Wt %**
**Academic stressors experienced while at University**	12438			7993			4445		
Time management issues/procrastination	8359	67.2	66.5	5810	72.7	72.2	2549	57.3	56.5
Problems achieving work/life/study balance	6901	55.5	54.4	5182	64.8	63.7	1719	38.7	38.0
Feeling too much pressure to succeed	6694	53.8	52.8	4394	55.0	53.9	2300	51.7	50.9
Exam anxiety	5954	47.9	47.3	3711	46.4	45.9	2243	50.5	49.7
Difficulty coping with study issues	5706	45.9	44.9	4077	51.0	50.0	1629	36.6	35.9
**Hours of paid work in past week**									
0	6753	46.2	46.5	3014	32.6	32.8	3739	69.6	69.5
1 to 20	5992	41.0	40.7	4597	49.7	49.4	1395	26.0	26.0
> 20	1880	12.9	12.8	1643	17.8	17.7	237	4.4	4.5
**Experienced financial difficulty while at university**									
Yes	3434	27.6	27.4	2491	31.2	30.8	943	21.2	21.4
No	9004	72.4	72.6	5502	68.8	69.2	3502	78.8	78.6
**Type of financial difficulty experienced in last 12 months**	13072			8345			4727		
Ran out of food and could not afford more	1328	10.2	10.1	948	11.4	11.2	380	8.0	8.2
Could not afford to buy medicine (prescribed or over the counter)	1245	9.5	9.3	964	11.6	11.2	281	5.9	6.0
Sometimes required to provide services in exchange of accommodation	1364	10.4	10.4	803	9.6	9.6	561	11.9	11.9
**Experienced homelessness**									
Yes	361	2.4	2.5	154	1.7	1.7	207	3.8	3.8
No	14391	97.6	97.5	9174	98.3	98.3	5217	96.2	96.2
**Experienced discrimination**	12452			8000			4452		
**At the university**	3251	26.1	25.5	1950	24.4	23.7	1301	29.2	28.8
**In the wider community**^a^	6565	52.7	51.2	4286	53.6	51.5	2279	51.2	50.5
Gender	3148	25.3	22.9	2592	32.4	29.5	556	12.5	11.3
Race	2935	23.6	23.6	1424	17.8	17.8	1511	33.9	33.8
Ethnicity	2151	17.3	17.2	1237	15.5	15.4	914	20.5	20.4
Religion	944	7.6	7.6	575	7.2	7.2	369	8.3	8.2
Sexuality	861	6.9	6.9	706	8.8	8.8	155	3.5	3.6
Another minority status	274	2.2	2.2	163	2.0	2.0	111	2.5	2.5
Other discrimination	401	3.2	3.1	307	3.8	3.7	94	2.1	2.1
**Ever afraid in an adult intimate relationship**^b^									
Yes	1875	22.5	21.4	1339	24.0	22.7	536	19.5	18.8
No	6457	77.5	78.6	4248	76.0	77.3	2209	80.5	81.2
**Experienced unwanted sexual contact**									
Yes	3741	30.5	28.5	3020	38.2	35.9	721	16.6	15.4
No	8509	69.5	71.5	4893	61.8	64.1	3616	83.4	84.6
**Experienced unwanted sexual contact in past 12 months**^c^									
Yes	769	12.3	11.8	576	12.6	12.2	193	11.4	11.1
No	5501	87.7	88.2	3994	87.4	87.8	1507	88.6	88.9
**Had sex when you did not want to in past 12 months**^c^									
Yes	769	12.3	11.8	576	12.6	12.2	193	11.4	11.1
No	5501	87.7	88.2	3994	87.4	87.8	1507	88.6	88.9
**Victim of forced sex**									
Yes	979	8.0	7.3	755	9.5	8.7	224	5.2	4.8
No	11281	92.0	92.7	7158	90.5	91.3	4123	94.8	95.2
**Victim of attempted forced sex**									
Yes	935	7.6	7.0	750	9.5	8.7	185	4.2	3.9
No	11331	92.4	93.0	7163	90.5	91.3	4168	95.8	96.1

^a^Experienced at least one form of discrimination of the seven forms listed below– responses are not mutually exclusive

^b^Total students who had been in an intimate adult relationship = 8,336; 5591 (60.5% of 8,042) Local students and 2745 (61.3% of 4,481) International students

^c^For students who reported having experienced intercourse (vaginal and/or oral) in the past 12 months (Total students with a response = 6270; 4,570 Local students and 1,700 International students)

### Academic stressors

Most students reported experiencing one or more types of academic stressor, including time management issues, problems achieving a work/life/study balance or difficulty coping with study issues, with more local students reporting experiencing these stressors than international students. About half of local and international students reported feeling too much pressure to succeed while at university or experiencing exam anxiety.

### Financial issues

About two thirds of local students and one third of international students were working in paid employment in the week preceding the survey with most working one to 20 h per week and a greater proportion of local students than international students working more than 20 h per week. Despite these levels of employment, around one in three local and one in five international students reported experiencing financial difficulty while at university. Moreover, approximately one in ten students had at times run out of food and could not afford to buy more, could not afford to buy medicine in the last 12 months, or were required to provide personal services (such as housework, care duties or sexual services) in exchange for accommodation. Of the local and international students who responded to the question, 1.7% (*n* = 154) and 3.8% (*n* = 207) respectively reported homelessness at some stage during the past 12 months.

### Cultural stress and discrimination

Just over half of all students reported experiencing at least one form of discrimination in the wider community and by contrast around one in four reported at least one form of discrimination while at university. The most common type of discrimination experienced in the wider community reported by local students was based on gender, and international students reported discrimination most commonly based on race and ethnicity.

### Abuse and violence

Non-consensual sexual experiences were common amongst the study sample. Around 7% of all students reported they had experienced forced or attempted forced sex (vaginal, oral, or anal) in their lifetimes. Amongst those who reported having intercourse (vaginal or anal) in the past 12 months, over one in ten local and international students reported having had sex when they did not want to. More than one in every four students reported other forms of unwanted sexual contact (e.g., uninvited touching or kissing of a sexual nature). The prevalence of these sexual experiences was approximately two times higher for local students than international students. Students were also asked about lifetime experiences of fear, abuse, and violence in current and past relationships. Of those who had ever been in an intimate adult relationship (*n* = 8332), over one in five students reported that they had ever been afraid of a partner and around 9% (result not in table) had been afraid in the previous 12 months.

#### Learning and academic factors

Of 6,704 local and 3,805 international students who had completed at least one subject in the first half of the 2019 academic year less than one in ten (7.8% (*n* = 508) and 10.1% (*n* = 373), respectively) failed one or more subjects. Students were asked if they had considered dropping out of their current course at any time in the past 12 months, and if so, students could select one or more reasons (Table 7). Over one quarter of students who answered this question had considered dropping out, with the top three reasons given by both local and international students being difficulties due to *health or stress*, *study/life balance*, and *difficulty with workload*. Although similar levels of concern among local and international students were noted for many reasons, there were some key differences: one in five local students indicated that *paid work responsibilities* or their *need to do paid work* had led them to consider dropping out (compared with approximately 5% of international students); for international students, *expectations not having been met* (26.4%), and *difficulty paying fees* (16.3%—result not in table) were more commonly identified as reasons to consider dropping out compared to local students (17.6% and 9.4% (result not in table), respectively).

**Table 7:** Learning and Academic Factors (*N* = 14,880) and by Local (*N* = 9,398) and International students (*N* = 5,482)

	**Total**			**Local**			**International**		
	**N**	**%**	**Wt %**	**n**	**%**	**Wt %**	**n**	**%**	**Wt %**
**Failed at least one subject in 2019 Semester 1**^a,b^									
No fails	9628	91.6	91.4	6196	92.4	92.2	3432	90.2	89.9
At least one fail	881	8.4	8.6	508	7.6	7.8	373	9.8	10.1
**Considered dropping out of current course in past 12 months**									
Yes	3353	27.0	26.6	2294	28.7	28.3	1059	23.8	23.7
No	9085	73.0	73.4	5699	71.3	71.7	3386	76.2	76.3
Most common reasons (from a list of 30 reasons^c^ for dropping out^d^)									
Health or stress	1824	54.4	53.3	1348	58.8	57.6	476	45.0	44.2
Study/life balance	1492	44.5	44.1	1156	50.4	49.9	336	31.8	32.0
Difficulty with workload	1464	43.7	43.1	1141	49.8	49.2	323	30.5	30.3
Need a break	1097	32.7	32.4	804	35.1	34.8	293	27.7	27.3
Personal reasons	953	28.4	28.5	684	29.8	30.0	269	25.4	25.4
Lack of academic support	863	25.8	25.9	562	24.5	24.6	301	28.4	28.8
Standards too high	744	22.2	21.8	526	22.9	22.4	218	20.6	20.6
Financial difficulties	742	22.1	22.2	534	23.3	23.2	208	19.7	20.1
Lack of interest	704	21.0	21.1	499	21.8	22.0	205	19.4	19.1
Expectations not met	686	20.5	20.5	403	17.6	17.6	283	26.7	26.4
Change of direction	681	20.3	20.8	523	22.8	23.5	158	14.9	15.1
Lack of career prospects	654	19.5	19.5	446	19.5	19.4	208	19.7	19.7
Need to do paid work	622	18.6	18.3	543	23.7	23.4	79	7.5	7.6
Paid work responsibilities	500	14.9	14.7	466	20.3	20.2	34	3.2	3.1

^a^Data reported only from students who consented to have their survey results linked to their academic performance transcripts

^b^Students who did not complete any subjects for Semester 1 were excluded from the denominator. Total number of participants that consented to accessing official academic records and had undertaken at least one subject in Semester 1 = 10,509; 6,704 local students and 3,805 international students

^c^Other reasons listed in survey: Boredom, family responsibilities, lack of administrative support, difficulty paying fees, gap year/deferral, quality concerns, lack of government assistance, social reasons, commuting difficulties, Other reasons, Travel or tourism, Other opportunities, moving residence, institution reputation, received other offer

^d^Reasons for dropping out are not mutually exclusive; Denominator used to calculate the percentages for the reasons were based on the number who considered dropping out. Note: two local students and one international student had missing responses for the reasons

#### Social and cultural environment

Students were asked about a range of contextual factors relating to their social and cultural environment (Table 8). The most common living arrangements reported by local students were either living with parents/relatives rent‐free or renting, whereas most international students were renting. Just under half of local students reported being in a current romantic relationship (dating, married or de facto) compared to about two in five international students. Fewer than one in three students reported being involved in an organised social, sporting or recreational group either at university or elsewhere.

**Table 8:** Social and Cultural Environment all students (*N* = 14,880) and by Local (*N* = 9,398) & International students (*N* = 5,482)

	**Total**			**Local**			**International**		
	**n**	**%**	**Wt %**	**n**	**%**	**Wt %**	**n**	**%**	**Wt %**
**Current living arrangements**									
Renting flat, apartment or house	8001	54.2	54.5	3385	36.3	36.3	4616	85.1	85.2
With parents/relatives, rent-free	4220	28.6	28.5	4044	43.4	43.5	176	3.2	3.2
University residence/college	996	6.8	6.7	611	6.6	6.5	385	7.1	7.1
Own/mortgaged home	1065	7.2	7.1	921	9.9	9.8	144	2.7	2.7
Boarding including paying rent to parents/relatives	354	2.4	2.4	302	3.2	3.3	52	1.0	0.9
Other	116	0.8	0.8	65	0.7	0.7	51	0.9	0.9
**Current relationship status**									
Single	8011	54.3	54.4	4775	51.2	51.3	3236	59.7	59.6
Dating	4479	30.4	30.2	2976	31.9	31.9	1503	27.7	27.5
Married/de facto	2171	14.7	14.8	1513	16.2	16.2	658	12.1	12.4
Other (Engaged, divorced, separated, widowed, undefined)	86	0.6	0.6	60	0.6	0.6	26	0.5	0.5
**Involved in organised groups at university or elsewhere**^a^	12429			7990			4439		
Social club or university group	3432	27.6	27.6	2121	26.5	26.6	1311	29.5	29.4
Sport or physical recreation group	2967	23.9	24.6	2229	27.9	28.7	738	16.6	17.4
Special interest/hobby group including online groups	1658	13.3	13.5	1150	14.4	14.6	508	11.4	11.4
**Average number of hours spent on social media on typical day across past 2 weeks**									
None	667	5.4	5.6	496	6.2	6.4	171	3.8	4.1
1—2 h	6525	52.4	52.8	4534	56.7	57.0	1991	44.7	45.6
3—5 h	4058	32.6	32.0	2394	29.9	29.5	1664	37.4	36.5
6 or more hours	1202	9.7	9.5	576	7.2	7.1	626	14.1	13.8
**Extent social media currently creates stress**									
Not at all	3315	26.6	27.3	2126	26.6	27.3	1189	26.7	27.4
A little	4078	32.7	32.6	2554	31.9	31.8	1524	34.2	34.0
Somewhat	3271	26.3	25.9	2073	25.9	25.6	1198	26.9	26.5
A lot	1431	11.5	11.4	973	12.2	12.0	458	10.3	10.2
A great deal	357	2.9	2.8	274	3.4	3.4	83	1.9	1.8
**Experienced any of the following while at University**^b^	12438			7993			4445		
Loneliness	5319	42.8	42.4	3453	43.2	42.9	1866	42.0	41.5
Family difficulties	3077	24.7	24.1	2370	29.7	28.9	707	15.9	15.7
Travelling/commuting difficulties	2878	23.1	22.8	2395	30.0	29.6	483	10.9	11.0
Relationship issues	2704	21.7	21.7	1823	22.8	22.7	881	19.8	19.9
Homesickness issues	1964	15.8	15.4	850	10.6	10.3	1114	25.1	24.3
Accommodation/living arrangements	1809	14.5	14.4	1052	13.2	12.9	757	17.0	17.1
	**Mean**	**SD**	**Wt Mean**	**Mean**	**SD**	**Wt Mean**	**Mean**	**SD**	**Wt Mean**
**Social support (MOS-SSS-6**)^c^	22.56	6.39	22.45	24.17	5.55	24.08	19.67	6.78	19.59
**Acculturative stress (SAFE)**^d^	18.89	13.22	18.98	15.36	12.18	15.45	25.08	12.70	25.04

^a^Activities are not mutually exclusive

^b^Experiences are not mutually exclusive

^c^Social support (MOS-SSS-6) [60] measured using the Medical Outcomes Study-Social Support Survey. Scores range from 6 to 30 with higher scores indicating greater perceived social support; Total students with a response = 12,478; 8,014 Local students and 4,464 International students)

^d^Acculturative stress is measured using the Social, Attitudinal, Familial and Environmental Acculturative Stress Scale (SAFE) scale [57], where higher scores are indicative of higher degrees of stress. Total students with a response = 13,443; 8,563 Local students and 4,880 International students

Most students reported using social media for up to two hours per day however more international students were using social media between three and five hours each day and 13.8% of international students reported spending six or more hours per day on social media platforms compared to 7.1% of local students. Moreover, social media use was reported to cause at least *a little* distress for around 70% of the overall sample, and approximately 14% reporting that it caused ‘*a lot’* or ‘*a great deal’* of stress.

The Medical Outcomes Study—Social Support Survey (MOS-SSS) has six individual items that describe having someone to: *help if confined to bed*; *take you to the doctor if needed*; *share private worries or fears with*; *help solve problems*; *do something enjoyable with; make them feel loved and wanted* [60]. Each item has 5 response options (1 = none of the time; 2 = a little of the time, 3 = some of the time, 4 = most of the time, 5 = all of the time). Aggregate scores range from six to 30 with higher scores indicating greater perceived social support. The overall mean MOS-SSS score in this sample was 23/30 with a higher mean score for local compared to international students (Table 8).

Around 40% of all students reported experiencing loneliness while at university. Family difficulties were experienced by more than one in four local students and around one in six international students. Higher proportions of international students experienced *homesickness* and *issues with accommodation or living arrangements* compared with local students while commuting difficulties were more problematic for local students.

Acculturation refers to the cultural and psychological change that occurs when individuals from two or more cultures are in contact; the process can be smooth or give rise to stress [77]. Acculturative stress in this study was measured using the 13-item Social, Attitudinal, Familial and Environmental Acculturative Stress Scale (SAFE) in which each item is rated on a five-point scale from 1 = not stressful to 5 = extremely stressful [57]. The overall score ranges from zero to 65 with higher score indicating higher levels of acculturative stress. The mean acculturative stress score was much higher amongst international students compared to local students (Table 8).

#### Health and wellbeing services

Study participants were asked to identify from a list of health, wellbeing and support services offered by the university, which they were aware of, and which they had used (Table 9). In addition, they were asked about their use of health services external to the university (Table 9).

**Table 9:** Health and Wellbeing Services for all students (*N* = 14,880) and by Local (*N* = 9,398) & International students (*N* = 5,482)

	**Total**			**Local**			**International**		
	**n**	**%**	**Wt %**	**n**	**%**	**Wt %**	**n**	**%**	**Wt %**
	12,405			7976			4429		
**Aware of at least one university service listed below**^a^	11576	93.3	93.0	7463	93.6	93.3	4113	92.9	92.6
General Health Service	7801	62.9	62.8	4786	60.0	60.0	3015	68.1	67.8
Academic Skills	8862	71.4	70.8	5819	73.0	72.3	3043	68.7	68.3
Counselling and Psychological Services	7615	61.4	60.7	5537	69.4	68.8	2078	46.9	46.4
Student Housing	5197	41.9	41.9	3546	44.5	44.4	1651	37.3	37.5
Financial Aid	5181	41.8	42.0	3671	46.0	46.2	1510	34.1	34.4
Free Student Health Checks	3587	28.9	29.2	2371	29.7	30.1	1216	27.5	27.7
Student Equity and Disability Support	4106	33.1	32.7	3231	40.5	40.1	875	19.8	19.8
Safer Community Program^b^	2832	22.8	22.8	1819	22.8	22.8	1013	22.9	22.9
Legal Services	2828	22.8	23.1	2032	25.5	25.8	796	18.0	18.2
International Student Support Team	2331	18.8	18.9	1160	14.5	14.6	1171	26.4	26.4
Student Advocacy Services^c^	1809	14.6	14.7	1390	17.4	17.6	419	9.5	9.6
Indigenous Student Support Team	1320	10.6	10.6	1137	14.3	14.3	183	4.1	4.2
**Accessed at least one of the university services listed below**^**a**^	7430	59.9	59.3	4288	53.8	53.1	3142	70.9	70.3
Academic Skills	3505	28.3	27.8	1891	23.7	23.2	1614	36.4	36.0
General Health Service	3034	24.5	24.1	1511	18.9	18.6	1523	34.4	33.6
Counselling and Psychological Services	1584	12.8	12.4	1080	13.5	13.2	504	11.4	10.9
Financial Aid	901	7.3	7.3	617	7.7	7.8	284	6.4	6.5
Student Equity and Disability Support	792	6.4	6.1	682	8.6	8.2	110	2.5	2.5
Free Student Health Checks	677	5.5	5.5	324	4.1	4.1	353	8.0	8.1
Student Housing	467	3.8	3.8	164	2.1	2.1	303	6.8	6.9
Legal Services	225	1.8	1.9	129	1.6	1.7	96	2.2	2.2
International Student Support Team	214	1.7	1.8	13	0.2	0.2	201	4.5	4.5
Student Advocacy Services	184	1.5	1.5	134	1.7	1.7	50	1.1	1.2
Safer Community Program	182	1.5	1.4	91	1.1	1.1	91	2.1	2.0
Indigenous Student Support Team	99	0.8	0.8	81	1.0	1.0	18	0.4	0.5
**Access to health and wellbeing services provided by the university in past 12 months**	12405			7976			4429		
General Health Service	2516	20.3	19.9	1179	14.8	14.5	1337	30.2	29.4
Mental Health Services	815	6.6	6.5	483	6.1	6.0	332	7.5	7.3
Dentist	331	2.7	2.7	213	2.7	2.7	118	2.7	2.7
**Access to health and wellbeing services external to the university in past 12 months**	12405			7976			4429		
General Health Service	7527	60.7	60.0	6264	78.5	77.9	1263	28.5	28.2
Mental Health Services	1987	16.2	15.4	1802	22.6	21.8	185	4.2	4.0
Dentist	5197	41.9	41.4	4647	58.3	57.9	550	12.4	12.2
**Needed mental or emotional care/support but could not access in past 12 months**^d^	3016	24.3	23.7	1927	24.2	23.5	1089	24.6	24.0
**Barriers experienced in accessing mental or emotional care/support services**^**e**^									
Cost	1437	47.6	46.9	997	51.7	51.0	440	40.4	40.0
Uncertainty about whom to see	1393	46.2	46.1	928	48.2	48.1	465	42.7	42.8
Decided not to seek care	1208	40.1	40.4	816	42.3	42.6	392	36.0	36.5
Personal or family responsibilities/too busy	999	33.1	32.4	755	39.2	38.4	244	22.4	22.0
Confidentiality/embarrassment	718	23.8	23.7	507	26.3	26.3	211	19.4	19.2
No appointments	661	21.9	21.4	468	24.3	23.7	193	17.7	17.3
Limited opening hours	659	21.9	21.5	453	23.5	23.2	206	18.9	18.5
Lack of awareness of available services	614	20.4	20.4	371	19.3	19.1	243	22.3	22.8
No service available when needed	513	17.0	16.6	353	18.3	18.0	160	14.7	14.3
Waiting time too long	275	9.1	9.0	186	9.7	9.6	89	8.2	8.0
No private health insurance	270	9.0	8.7	216	11.2	11.0	54	5.0	4.8
Language problems	253	8.4	8.4	26	1.3	1.3	227	20.8	20.5
Transportation problems	205	6.8	6.7	172	8.9	8.8	33	3.0	3.1
Not taking new patients	190	6.3	6.0	165	8.6	8.2	25	2.3	2.2
Trouble understanding the terms used by health care professional	126	4.2	4.2	45	2.3	2.4	81	7.4	7.3
Cultural/religious reasons	46	1.5	1.5	26	1.3	1.3	20	1.8	1.9
**Online services accessed for own health**	12405			7976			4429		
Australian	6139	49.5	48.7	4676	58.6	57.7	1463	33.0	32.3
Home country	1893	15.3	15.2	521	6.5	6.6	1372	31.0	30.5

^a^Responses are not mutually exclusive

^b^Safer Community Program provides advice and support to members of the University community about their safety, and offers a central point of inquiry and reporting for inappropriate, concerning or threatening behaviour

^c^Student Advocacy Services offers students assistance and support for concerns such as assessment disputes, grievances, bullying, discrimination, sexual harassment and intellectual property

^d^Total students with a response = 12,389; 7,963 local and 4,426 international students

^e^Denominator is the number who had experienced one or more barriers accessing services/care in the past 12 months; Discrepancies in total due to missing responses

The university services known to the largest number of students were those addressing academic skills, counselling and psychological services and primary care. Overall, fewer international students were aware of the broad range of support services available at the university compared with local students, except for the International Student Support team. For example, while over two thirds of local students were aware of the availability of counselling and psychological services, fewer than half of international students knew of these services. A greater percentage of international students compared to local students accessed on-campus university services, predominantly the academic skills unit and the primary care service.

For the participants who provided a response, access to health service providers external to the university in the previous 12 months was greater amongst the local students compared to the international students (Table 9). Specifically, nearly 80% of local students compared to 28.2% of international students accessed primary care through general practitioners (GPs), and 57.9% of local and 12.2% of the international students accessed dentists.

Access to online health information and or services (such as assessment tools, mental health self-help programs, apps for mental and general health, calling crisis helpline or online chat support, health chatrooms or support groups) was common in the sample (Table 9). Accessing Australian online information and services was more common than international sites, however 30.5% (*n* = 1372) of international students reported using online services in their own home country.

Despite the array of health services available to students, whether on campus, in the general community, or online, almost one quarter of students indicated that there were times in the previous 12 months when they needed mental or emotional support but could not get it (Table 9). By contrast, less than one in ten students (6.7% of the 7962 local and 10.0% and 4,426 international students who responded to the item) indicated they needed general health care but could not get it (result not shown in Table 9). Students were asked to identify from a list any barriers they experienced in accessing mental or emotional care or support services. The most common barrier to accessing both mental health and general health services was *cost* and *uncertainty about whom to see.* Other common barriers included *limited opening hours*, and *no appointments*. For local students, *personal or family responsibilities/too busy* was also a factor, whereas for international students *lack of awareness of available services* and *language problems* were also a concern.

## Discussion

This paper describes the method and selected results of a comprehensive survey designed to quantify the prevalence of a wide variety of factors that may impact on university student mental health, wellbeing, and academic performance. The study also provides a baseline for a follow-up of the cohort which has been undertaken during the COVID-19 pandemic. Findings from future analyses of risk factors for poor mental health and academic outcomes will inform a whole of university approach to promoting the wellbeing of all students. We reported on the eight survey domains by local and international student citizenship status to understand whether mental health promotion and management strategies would require tailoring for either group. While we have reported on prevalence of several health and wellbeing factors, we discuss here only key findings for prevalence of mental health and social issues and health service access. Subsequent papers will report the effects of each domain (such as social media use, violence, risk taking behaviours) on mental health and academic outcomes.

### Mental health issues

A key finding of our study is that almost one in every three students reported experiencing psychological distress (defined as experiencing ‘moderate’, ‘moderately severe’ or ‘severe’ depressive symptoms on the PHQ-9) and around 23% reported moderate to severe anxiety in the previous 2 weeks. The prevalence of mental disorders identified in our study is similar to rates reported for tertiary student populations in other countries around the world [23, 78, 79]. Across the eight countries (Australia, Belgium, Germany, Mexico, Northern Ireland, South Africa, Spain, USA) included in the *World Mental Health – International College Students* study, the 12‐month prevalence estimates for any mental disorder ranged from 19.1% in Belgium to 43.3% in Australia [23]. Another review of prevalence rates for psychological distress in university students globally reported rates of around one in ten in Nigeria and China, approximately one in five in Denmark, Norway and Japan, more than one in four in France and Turkey, and more than a third in Poland and Bulgaria [80].

The high prevalence of mental health problems and probable depression and anxiety reported in this study is not surprising given that university students are mostly in an age range that is the peak period of onset of mental and substance use disorders [81]. However, there seems to be a lack of consensus within the literature about whether university students experience the same or greater mental distress than young people of similar age who are not attending university [10, 80, 82]. This is important to establish when ascertaining whether attending university places an additional mental health burden on young people. Some scholars suggest that the issue which distinguishes university from non‐university same-aged peers is their greater need to balance the competing demands of work, life, and study pressures [30], although more analyses are required to understand the interplay between all these individual student factors and their impact on mental health and wellbeing. Our finding that one in three students had experienced moderate to severe distress is higher than the general population and echoes previous Australian research measuring the prevalence of psychological distress among tertiary students and non‐students using population‐representative data from two national surveys [10]. Higher prevalence of moderate distress was reported in students compared with non-students in both the 2007 Household, Income and Labour Dynamic in Australia (HILDA) survey (27.1% vs 21.2%) [83] and in the 2007 National Survey of Mental Health and Wellbeing (27.4% vs 19.5%) [82], however, there was no difference in the prevalence of high distress [10]. It should be noted that these surveys are now over a decade old and may not be reflecting contemporary Australian population prevalence. A more definitive conclusion would be possible if future national studies of mental health in young people asked respondents about attendance at university, and if there was greater consistency amongst survey instruments used in research.

Our study also revealed a higher prevalence of eating disorders (21.2%) compared with the general population. Over 16% of the Australian population eare affected by eating disorders and disordered eating together [84]. Our survey used a screening instrument called the SCOFF which indicates probability of an eating disorder, some of which may not be confirmed on full assessment. This might explain the higher prevalence of disorder in our sample. However, it is worth noting that our prevalence of probable eating disorder was also higher than a US study of college students where the SCOFF was similarly utilised. The US study reported rates of 9.4% amongst undergraduates, and 5.8% amongst graduates [64].

The prevalence of some mental health issues in our study was a little higher amongst local students than international students. For example, self-reported mental health concerns; prevalence of moderate to severe depressive and anxiety symptoms as measured by the PHQ-9 and GAD-7 respectively; and proportion who have tried to self-harm in the previous 12 months. Probable eating disorder, on the other hand, was more prevalent amongst international students and the measure on resilience indicates slightly higher levels amongst local students. Under-reporting of mental health concerns by international students is possible; for example Asian international students reportedly seek help for psychological problems less frequently than local students [85] due to perceived stigma about mental health service use [28, 85, 86].

Risk-taking behaviours which may affect and be affected by mental health concerns such as hazardous drinking and use of other drugs [58, 87, 88] had a much higher prevalence amongst local students than international students in our sample. Other research also reports international students being less engaged in these health risks [24].

Annual surveys in the US have documented an increase in prevalence of depression, suicidal ideation and self‐harm for the general population of adolescents and young people [89] while studies of university students in the US show a trend toward increasing prevalence of mental disorder year-by-year over the last decade [90]. In addition, a review of studies of attendees to university counselling services has revealed a greater severity and complexity of presenting mental health issues when compared to earlier studies in the late 1980s [80]. Reasons for this apparent rise in prevalence of mental health issues amongst university students over time are not clear.

### Psycho-social issues

It is well-recognised that mental health conditions are often underpinned by other psychosocial problems, for example, academic stressors were associated with distress in a review of university student studies [80]. In Australia, number of hours spent studying, either low or high amounts, was associated with mental ill‐health symptoms [30] especially where students felt unsupported by teachers and their faculty, and where they were not satisfied with their course [91]. Researchers have also found that while ethnicity, gender, and place of birth were not uniquely related to mental health, juggling work/life responsibilities with academic work was associated with poorer mental health [80]. Certainly, in this present study, the balancing of competing demands (work/study/life) was a significant stressor and reason for considering dropping out of studies for both local and international students, along with feeling pressure to succeed, exam anxiety and stress due to time management, and difficulty coping with study issues. A small longitudinal study examined students entering university from high school and noted the rise in mental disorder when measures were repeated in first year university, even when there were no symptoms in high school [92]. This highlights the significant life transition and challenges to mental health that entering university brings [80].

Over one quarter (27.4%) of both local and international students in our study experienced financial difficulties, including not being able to afford food (10.1%) or prescription medicines (9.3%). A greater proportion of local students worked in excess of 20 h per week, however this may be because international student visas can preclude employment. Some students have had to provide services in exchange for accommodation (10.4%) and a small proportion of students (2.5%), more international than local, have experienced homelessness. The review by Sharp and Theiler [80] reveals that financial concerns, including those amongst Australian samples, are significant for university students compared to non‐student peers, and that these concerns are associated with psychological symptoms. One study in the review reported that 20% of students experience financial crisis or go without food which compares with our study. In a longitudinal study in the review [92], financial hardship significantly contributed to later depression. Being in paid work has also been found to be associated with symptoms of mental ill‐health when more than 15 h per week of work was undertaken [91]. These issues highlight that while university students may be considered a privileged group because they are in higher education, they do experience socio-economic and other hardships which impact wellbeing.

Discrimination was experienced by both local and international students, mainly external to the university, for different key reasons; based mainly on gender for local students and on race and ethnicity for international students. Acculturative stress, also measured in our study, and perceived discrimination are strongly correlated with each other [93] and associated with depression [94]. The circumstances in which discrimination is experienced must be the subject of further in‐depth research to inform strategies and programs to reduce these forms of behaviour.

Non-consensual sexual experiences are frequently associated with poor mental health [95]. The National Student Safety Survey [96], undertaken in 2017 and 2021 across university campuses, confirms that sexual violence remains a serious issue for Australian students, both local and international. Its most recent iteration suggests that 4.5% of students had been sexually assaulted since starting university and 16% had been sexually harassed. Whilst these findings are important for institutions seeking to reduce rates of sexual violence on campuses, reporting on recent experiences only provides one piece of the puzzle. The trauma caused by sexual violence tends to be long-lasting [95, 97, 98], and hence from a wellbeing perspective it is also valuable to understand the lifetime prevalence of these experiences. Our survey addressed this gap by examining lifetime experiences of forced sex, attempted forced sex, or unwanted sexual contact. In addition, our sample included both undergraduate and graduate students, whereas most of the extant literature focuses only on undergraduates [99]. Our study hence provides a more comprehensive picture of the prevalence of sexual violence experiences across an entire campus. Our finding that 28.5% of students had ever been a victim of unwanted sexual contact, 7.3% had experienced forced sex and 7.0% experienced forced sex attempts is concerning. Yet, this is still likely to be an under-representation of the true scope of the problem. Students were not specifically asked about sexual acts obtained when incapacitated, unconscious or asleep. Alcohol and substances can play a large role in sexual assaults on college campuses [100], and students may not have understood an experience of “non‐consent” as one of being “forced”. A further consideration is that our study asked about specific sexual assault behaviours rather than asking students to define their experiences as “sexual assault”. Indeed, other studies in similar high‐income countries examining lifetime sexual violence experiences in university student cohorts [101–103] report varied prevalence rates depending on how questions were asked. Sivertsen and colleagues [103] found that 3.4% of a cohort of  over 50,000 students in a Norwegian sample had experienced “rape”, 2.1% had experienced a “rape attempt”, and 24% had experienced some other form of”sexual harassment”. On the other hand, Carey and colleagues utilised the Sexual Experiences Survey [104], which describes behaviours rather than using labels. They reported that 21% and 26% of a sample of first‐year US college women had experienced behaviours they classified as “incapacitated rape” or “forcible rape” respectively; the high proportions possibly explained by the inclusion of sexual violence where alcohol and substance use were a factor, and because they only surveyed women. Further exploration of contextual factors associated with the relatively high prevalence of life‐time sexual violence in this study will be undertaken in the future.

Little attention has been paid to the intimate partner violence (IPV) experiences of university students compared to sexual assault [105]. This is problematic given the overlap between these two forms of violence [106], and the elevated risk of IPV victimisation for the 18‐24 age group [107]. In our study, nearly a quarter of students who had been in a relationship had ever been afraid of a partner, with nearly 9% experiencing fear of partner in the previous 12 months. Although only those students who had been fearful of a partner were asked the Composite Abuse Scale questions, it is worth noting that over 5% of the entire sample indicated experiencing behaviours in the “severe combined abuse” category, which encompasses serious physical, psychological and sexual abuse. These findings highlight that lifetime experiences of IPV are pertinent for Australian university students, foregrounding the need for universities to include IPV in violence prevention programs and interventions alongside sexual assault. Given that both IPV and sexual violence experiences are consistently associated with depression [108, 109], campus services should be aware that these issues potentially underlie poor mental health presentations. Whether or not a student’s experience of sexual violence or IPV occurred during their candidature, universities have a responsibility to provide trauma‐informed services and support.

### Service access and support

International students overall reported lower social supports and awareness and use of health services compared to local students. This may considerably disadvantage their ability to cope with stressors. Addressing the barriers to accessing mental health care for international students was a key recommendation made by the Victorian Coroner in a review of suicides amongst international university students in Victoria [110]. Providing better mental health promotion, detection and management of disorder amongst students at university was also a recommendation of the recent Australian Productivity Commission into Mental Health [111].

The most common barriers to accessing general and mental health services reported in our study was cost which has also been highlighted as a major barrier in recent studies of vulnerable groups in Australia [112]. Primary care in Australia is covered by national health, but psychological services are only partly covered. International students require private insurance to pay for all health care in Australia. University psychological services do not charge a fee, however may be limited in capacity to cope with all students in need. These challenges require universities to consider the best ways to provide needed mental health services including links with external providers and online options [9].

Our data suggest that online approaches also have potential for engagement of students in mental health promotion or care with at least one in ten accessing interventions online and over three quarters accessing information online. The challenge will be in co‐design of these interventions with international students to properly engage them and link them with local supports, given that around a third of international students in this study go to their home country online services. A systematic review and meta-analysis of internet-based mental health interventions (2019), especially those modelled on cognitive behavioural therapy, concluded that these appear effective however should be developed, piloted and trialled with students from faculties beyond health alone, as health students may be more orientated to these interventions [113].

### Implications for mental health promotion in universities

There is little available evidence to guide institutional policy for improving mental health and suicide prevention, although some strategies show promise such as curriculum and assessment changes (e.g. introducing wellbeing courses and decreasing the number of graded assessments), and ‘gatekeepers’ who detect risk and connect students with services [114]. Universal mental health and wellbeing promotion, which addresses other psychosocial stressors (e.g. financial and housing stress) and which build protective factors (e.g. social connections, stress management, ways to balance life/study, curriculum assessment changes [114]) may also help reduce the number of students needing mental health care [115]. There is also a need for more detailed analyses of the factors that combine to indicate higher risk groups or situations and to understand which interventions work best for which students [113].

Reviews for primary suicide prevention [116], reduction of mental health stigma [116], and body image and eating disorders [117] on campus did not highlight approaches which had consistent results, hence more work is required in these areas. Interventions addressing physical activity, nutrition, and weight [118] and alcohol use [118] have been shown in systematic reviews to have robust evidence of benefit and should be examined by universities and if appropriate implemented and evaluated in each university setting.

### Strengths and limitations

There are several strengths in this study. We adopted a systematic approach to our survey design including: a co-design element with students; input from university stakeholders within chancellery, wellbeing services, residential colleges, and academic units; and a comprehensive literature search for measures that contributed to poorer or better outcomes in university students. Our online survey employed privacy measures to ensure researchers could not identify students and students’ responses could not be seen by university staff to assist in encouraging participation and disclosure of sensitive issues.

Our overall response rate of 28% was higher than most university student surveys, which typically achieve around 12% [47, 119]. Furthermore, 85% of our respondents agreed to linkage of their survey results with academic transcripts, for analysis of impact on academic outcomes in future work. The eight-country study of mental health amongst first-year college students carried out by the World Mental Health – International College Students group [22] used an online survey method and achieved a sample size of 13,984 with variable response rates across countries; Australia being the lowest (Australia 7.0%; South Africa 12.9%; Spain 13.0%; Germany 13.4%; USA 16.9%; Northern Ireland 17.0%; Belgium 53.7%; Mexico 79.3%) [23]. A recent Australian study of university students using a random sampling frame achieved a response rate of 11.6% (*n* = 611 students) [29].

Our respondents were largely representative of the university population, across all faculties, undergraduate, postgraduate and research higher degree students and in demographic characteristics such as local and international student status, gender, and age. The health and wellbeing factors we chose to measure in this university student population have been investigated previously [47] and were similar to those found in recent studies in other Australian university student populations [29, 32]. However, other studies do not report on their denominator data or the representativeness of their student sample, leaving less confidence in their prevalence estimates [23, 29, 30].

Despite our higher response rate compared to similar studies, and the representativeness of the sample population on demographic variables, there is still a risk that voluntary participation may have introduced a bias in the sample that we have not measured. Interpretation of findings therefore needs to keep this potential bias in mind. We had considered a smaller sample however once stratifying for important demographic variables that may influence the student experience such as age, gender, year of study, citizenship status, full or part-time, and faculty, it was a stronger design with minimal extra effort to maximise participation of the whole sample so that data could be examined for many characteristics and prevalence estimated. Our participant recruitment reminder system was effective at achieving this.

Although we aimed for our survey to include all areas which previous studies have identified as being relevant, and some newer areas such as stress induced by use of social media, there were some topics that we could not examine to keep the completion time within a manageable limit. These include risk exposures common in this age group such as road safety, sun protection, and gambling [120, 121] and more protective factors or indicators of positive wellbeing [122, 123]. We may have missed identifying potential groups at risk by not seeking information about students who were the first in their family (first generation) to attend university [124], their socioeconomic background [125], local students who needed to move away from home to attend university [126], and parental education or occupation [127]. Other limitations include that those with mental health concerns may be more or less likely to participate, creating a potential selection bias. Furthermore, this study was conducted in a large, high-ranking Australian university and while findings are similar to other universities nationally and globally, they may not be generalisable to other forms of higher education.

## Conclusion

Providing better mental health promotion, detection and management of disorders amongst students at university is a key recommendation of the recent Australian Productivity Commission into Mental Health (2019) [111] and subsequent proposed frameworks [128]. Our data provide information on prevalence of risk and protective factors for mental health and academic outcomes and on awareness and use of a variety of services both on and off campus to support students. These data will enable universities to formulate the goals of their health promoting frameworks. Universities also need to formulate an evaluation framework which documents health and wellbeing policy initiatives and their implementation, and routinely surveys student mental health indicators to track improvements. This will be especially important in the wake of COVID-19 where knowledge of the impacts on students’ learning and wellbeing is still evolving.

## Supplementary Information

**Additional file 1: Supplementary Appendix 1.** Derivation of scale scores from selected measures employed in student survey.

**Additional file 2: Supplementary Appendix 2.** Detail on methods for creating weighted percentages to account for potential response bias in the sample.

**Additional file 3.**

**Additional file 4.**

**Additional file 5: Supplementary Table 1.** Number and percentage of student sample who completed each section of the survey.

**Additional file 6: Supplementary Table 2.** Demographic characteristics of university population (*N*=56,392^1^) and by survey respondents (*N*=14,880) versus non-respondents (*N*=41,512).

## Data Availability

The data generated from the study is not publicly available due to the need to ensure participant privacy in accordance with the consenting procedures. Researchers interested in further study of the data may contact the authors regarding data access protocols.

## Abbreviations

PHQ-9: Patient health questionnaire 9 items used to assess for major depression
GAD-7: Generalised anxiety disorder 7 item scale
