# Supplementary material for: Towards a health promoting university: descriptive findings on health, wellbeing and academic performance amongst university students in Australia
Source: BMC Public Health. 2022 Dec 27;22:2430. doi: 10.1186/s12889-022-14690-9 (PMC9792939; doi:10.1186/s12889-022-14690-9)
Supplement: Supplementary file 1 — Additional file 1: Supplementary Appendix 1. Derivation of scale scores from selected measures employed in student survey. [file 12889_2022_14690_MOESM1_ESM.docx]

Supplementary Appendix 1: Derivation of scale scores from selected measures employed in student survey

Depression severity was examined using the *Patient Health Questionnaire-9* (PHQ-9) (41) which assesses the frequency of depressive difficulties experienced in the preceding 2-week period. The scale comprises nine items, with response options ranging from 0 to 3 (*Not at all* = 0, *Several days* = 1, *More than half the days* = 2, *Nearly every day* =3). Total scale scores range from 0 to 27 with higher scores indicating higher severity of depressive symptoms and a score of more than 10 indicating probable major depression.

Anxiety severity was assessed using the *Generalized Anxiety Disorder 7-item* (GAD-7) (43) that captures incidence of anxiety symptoms experienced in the previous 2 weeks. The 7-item scale responses range from 0 to 3 (*Not at all* = 0, *Several days* = 1, *More than half the days* = 2, *Nearly every day* =3). The total score ranges from 0 to 21 with higher scores indicating higher severity of anxiety symptoms.

Eating disorder was measured using the 5-item *Sick Control One stone Fat Food* (SCOFF) which assesses respondents’ eating behaviour patterns (42, 129). Each question in the scale is presented with 2 options (*Yes* = 1, *No* = 0); aggregate scores range from 0 – 5. An answer of *yes* to two or more questions indicates a potential case of anorexia or bulimia (42, 129).

Resilience was measured using the 10-item *Connor–Davidson Resilience Scale* (CD-RISC) which evaluates perceived ability to cope with adverse events (44, 63). Responses for each item (e.g., “I am able to adapt to change”) range from 0 to 4 (0 = *not true at all*, 1 = *rarely true*, 2 = *sometimes true*, 3 = *often true*, 4 = *true nearly all the time),* with an overall aggregate score between 0 and 40; higher scores reflect higher resilience.

To assess for heavy drinking and alcohol dependence the 3-item Alcohol Use Disorders Identification Test (AUDIT-C) (64, 130) was used. This instrument assessed the typical alcohol intake frequency and quantity, as well as binge drinking patterns in the previous year. Each item presented 5 options that ranged from 0 – 4 with an overall possible score of 0 to 12 (sum of 3 item scores). Hazardous drinking was indicated by a score of 3 or more on the AUDIT total score (64).

The Composite Abuse Scale (CAS) (40) was used to detect intimate partner violence, with questions assessing experiences in adult intimate relationships. The scale includes screening questions that assess whether the respondent has been afraid of a partner in the previous 12 months or has ever been afraid of a partner. In case of a positive response, 30 questions with five response options (*Never* = 0, *Only once* = 1, *Several times* = 2, *Once per month* = 3, *Once per week* = 4, *Daily* = 5) are next presented. The CAS comprises four subscales: Physical abuse (range: 0 – 35), Emotional Abuse (range: 0 – 55), Harassment (range: 0 – 20), Severe combined abuse (range: 0 – 35). Higher scores indicate higher levels of abuse.

Acculturative stress was measured using the 13-item Social, Attitudinal, Familial and Environmental Acculturative Stress Scale (SAFE) (68). Respondents were asked to rate each item on a 5-point scale from *not stressful* (1) to *extremely stressful* (5). If the item was not relevant, participants were requested to choose *not applicable* (0). Aggregate scores range from 0 to 65 with higher scores indicating higher levels of acculturative stress.

Students’ learning styles were assessed using a brief version of the Adelaide Diagnostic Learning Instrument (131, 132). The 21-item ADLIB explores student approaches to learning and comprises three subscales: *Distracted Learning*, representing disorganised time management, distraction and low enthusiasm; *Anxious/Inefficient Learning*, assessing poor organisation, excessive and ineffective effort, and worry about poor performance; and *Independent/Deep Learning*, capturing efforts to integrate ideas, relate ideas to evidence, and use questioning against evidence. All items are answered using a 4-point Likert scale (ranging from *strongly disagree (*0) to *strongly agree* (3)) with item scores summed to give subscale ranges of 0 – 21, 0 – 21, and 0 – 18, respectively. Higher scores in each subscale indicate a greater propensity towards that factor. Internal reliability of the subscales has been reported as acceptable (Cronbach alpha between 0.75 and 0.87) (69). In the present study a 20-item version of the ADLIB was used, with one item removed that was specific for health sciences students.

Social support was measured using the 6-item Medical Outcomes Study Social Support Survey (MOS-SSS) (58). The scale was used to assess various domains of perceived social support including tangible, emotional, positive social interaction and affectionate support. Each item is presented with 5 response options ranging from 1 to 5 (1 = *None of the time*, 2 = *A little of the time*, 3 = *Some of the time*, 4 = *Most of the time*, 5 = *All of the time*). Aggregate scores range from 6 to 30 with higher scores reflecting higher perceived social support.
