# Supplementary material for: Towards a health promoting university: descriptive findings on health, wellbeing and academic performance amongst university students in Australia
Source: BMC Public Health. 2022 Dec 27;22:2430. doi: 10.1186/s12889-022-14690-9 (PMC9792939; doi:10.1186/s12889-022-14690-9)
Supplement: Supplementary file 2 — Additional file 2: Supplementary Appendix 2. Detail on methods for creating weighted percentages to account for potential response bias in the sample. [file 12889_2022_14690_MOESM2_ESM.docx]

**Supplementary Appendix 2: Detail on methods for creating weighted percentages to account for potential response bias in the sample.**

To adjust for potential sampling bias we provided weighted percentages and means calculated using inverse probability weights (IPW) using Stata’s *survey* command. We first predicted each student’s probability weight of response by fitting a logistic regression on all the students who were sent an invitation to complete the survey. The dependant variable (outcome) was a binary response of whether the student responded to the survey (1) or not (0) and the predictor variables were gender (male/female/self-described) and citizenship (local and international students).

We identified the predictor variables by comparing summary statistics of available demographic characteristics on the university students (such as, age, gender, citizenship status, country of birth (Australia, China, Other), course level, faculty, fee status, attendance type, disability status) who were sent an invitation to the survey by whether they responded to the survey or not. The demographic profile and enrolment status of the 14,880 students who responded to the survey and were included in the analysis was similar to the 41,512 non-responders (see Supplementary Table 2), including mean age (24.8 (SD=6.9) vs 24.8 (SD=7.2), respectively). The exception was that females were more likely to respond than males (64% responded vs 54% males). Although citizenship was balanced between responders and non-responders, we included it as a predictor in the logistic model to calculate the IPW because the examination of local and international student issues was a key focus of this study.

To keep the identity of student responders private from researchers and the students’ survey responses private from university administrative staff, the university records used to compare the demographic characteristics for responders and non-responders could not be linked to the student’s survey responses. University administration were therefore only able to provide the summary statistics comparing responders’ and non-responders’ demographic characteristics and the predicted response weights for responders only. There was less than 1% discrepancy in the percentages in the demographic characteristics of responders between the university records and the self-reported survey data, indicating minimal response bias in the survey for the selected demographics characteristics. Also, Supplementary Table 2 included an additional 17 students (0.04%) of the 41,512 non-responders as they were recorded as being less than 18 years old according to university records. We could not identify these individuals in the university records to exclude them from the descriptive analysis provided in Supplementary Table 2. However, as they make up less than 0.04% of the non-responders they will not impact the weights. Generally, there was negligible difference between the sample and weighted summary statistics reported in Tables 3 to 9.
