# Supplementary figures and images for: Towards a health promoting university: descriptive findings on health, wellbeing and academic performance amongst university students in Australia
Source: BMC Public Health. 2022 Dec 27;22:2430. doi: 10.1186/s12889-022-14690-9 (PMC9792939; doi:10.1186/s12889-022-14690-9)

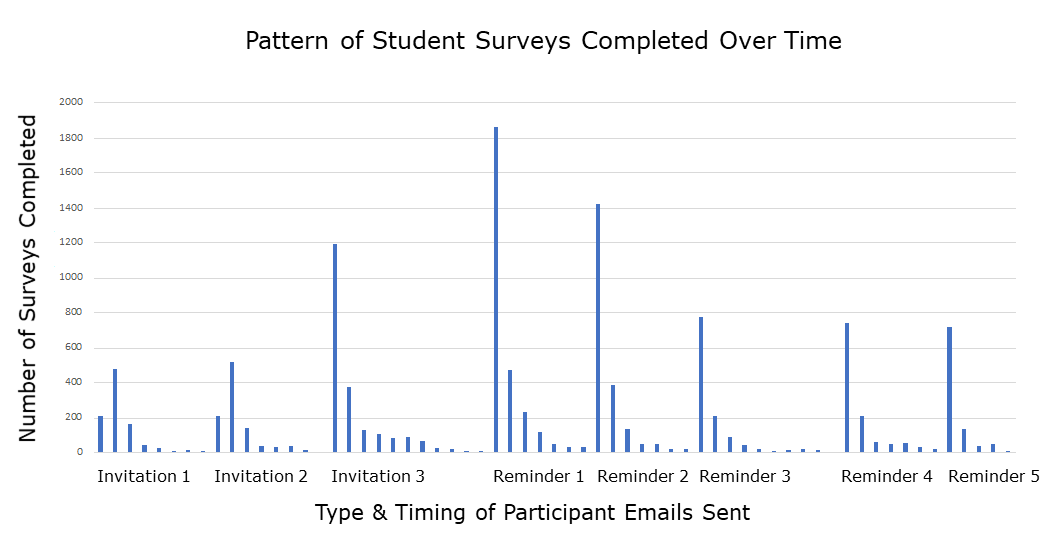

Supplement: Supplementary file 3 — Additional file 3. [file 12889_2022_14690_MOESM3_ESM.png]

# Survey domains

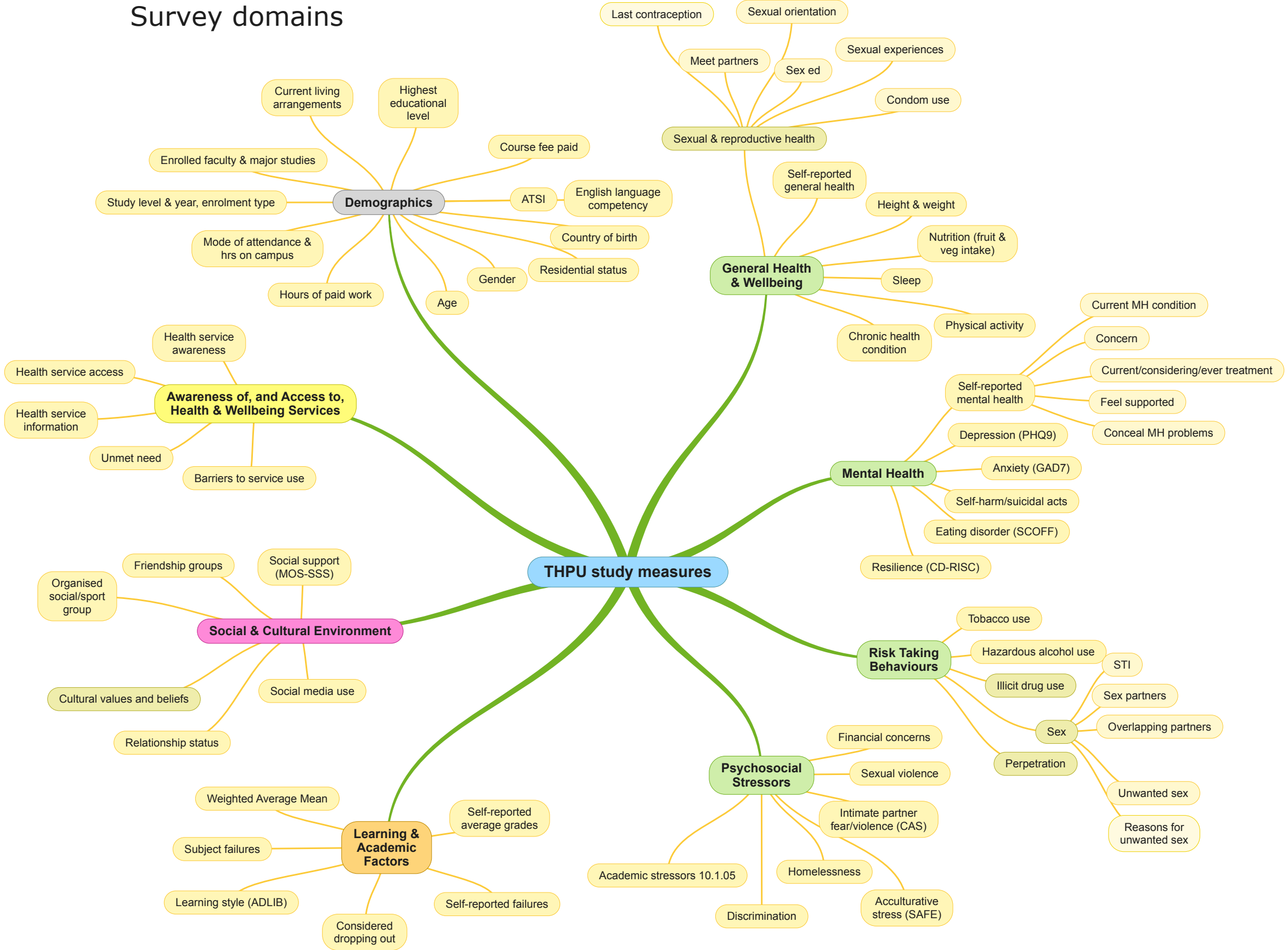

Supplement: Supplementary file 4 — Additional file 4. [file 12889_2022_14690_MOESM4_ESM.pdf]
