# Supplementary material for: Towards a health promoting university: descriptive findings on health, wellbeing and academic performance amongst university students in Australia
Source: BMC Public Health. 2022 Dec 27;22:2430. doi: 10.1186/s12889-022-14690-9 (PMC9792939; doi:10.1186/s12889-022-14690-9)
Supplement: Supplementary file 5 — Additional file 5: Supplementary Table 1. Number and percentage of student sample who completed each section of the survey. [file 12889_2022_14690_MOESM5_ESM.docx]

Supplementary Table 1: Number and percentage of student sample who completed each section of the survey

| **Section completed** | **n** | **(%)** |
| --- | --- | --- |
| General questions^1^ | 14390 | (96.71) |
| Access to academic records | 13931 | (93.62) |
| Learning Styles | 13443 | (90.34) |
| Cultural values and beliefs (part 1) | 13133 | (88.26) |
| Cultural values and beliefs (part 2) | 13072 | (87.85) |
| General Well-being (part 1) | 13004 | (87.39) |
| General Well-being (part 2) | 12871 | (86.50) |
| Psychological Distress (part 1) | 12782 | (85.90) |
| Psychological Distress (part 2) | 12747 | (85.67) |
| Risk-taking Behaviours (part 1) | 12734 | (85.58) |
| Risk-taking Behaviours (part 2) | 12694 | (85.31) |
| Risk-taking Behaviours (part 3) | 12587 | (84.59) |
| Risk-taking Behaviours (part 4) | 12547 | (84.32) |
| Experience of Violence (part 1) | 12538 | (84.26) |
| Experience of Violence (part 2) | 12522 | (84.15) |
| Experience of Violence (part 3) | 12520 | (84.14) |
| Experience of Violence (part 4) | 12478 | (83.86) |
| Social support and Resilience (part 1) | 12460 | (83.74) |
| Social support and Resilience (part 2) | 12452 | (83.68) |
| Healthy Places and Health Service Awareness (part 1) | 12405 | (83.37) |
| Healthy Places and Health Service Awareness (part 2) | 12347 | (82.98) |

^1^ Although 14,880 students commenced the survey a reduced number completed the first section
