# Supplementary material for: Towards a health promoting university: descriptive findings on health, wellbeing and academic performance amongst university students in Australia
Source: BMC Public Health. 2022 Dec 27;22:2430. doi: 10.1186/s12889-022-14690-9 (PMC9792939; doi:10.1186/s12889-022-14690-9)
Supplement: Supplementary file 6 — Additional file 6: Supplementary Table 2. Demographic characteristics of university population (N=56,3921) and by survey respondents (N=14,880) versus non-respondents (N=41,512). [file 12889_2022_14690_MOESM6_ESM.docx]

Supplementary Table 2: Demographic characteristics of total university population (N=56,392) and by survey respondents (N=14,880) versus non-respondents (N=41,512)

|  | **University population**^1^ | | | |  | |
| --- | --- | --- | --- | --- | --- | --- |
|  | **Total**  (56,392)^1^ | | **Survey respondents**  (14,880) | | **Survey non- respondents**  (41,512) | |
|  | **n** | **%** | **n** | **%** | **n** | **%** |
| **Gender** |  |  |  |  |  |  |
| Male | 24318 | 43.1 | 5305 | 35.7 | 19013 | 45.8 |
| Female | 31998 | 56.7 | 9551 | 64.2 | 22447 | 54.1 |
| Self-described | 74 | 0.13 | 24 | 0.16 | 50 | 0.12 |
| **Citizenship** |  |  |  |  |  |  |
| Domestic | 35121 | 62.3 | 9289 | 62.4 | 25832 | 62.2 |
| Overseas | 21271 | 37.7 | 5591 | 37.6 | 15680 | 37.8 |
| **Country of Citizenship** |  |  |  |  |  |  |
| Australia | 31364 | 55.6 | 8332 | 56.0 | 23032 | 55.5 |
| China (excludes SAR^2^ and Taiwan) | 13402 | 23.8 | 2686 | 18.1 | 10716 | 25.8 |
| Other or unknown | 11626 | 20.6 | 3862 | 26.0 | 7764 | 18.7 |
| **Course level** |  |  |  |  |  |  |
| Undergraduate | 27948 | 49.6 | 7000 | 47.0 | 20948 | 50.5 |
| Masters (coursework) | 23067 | 40.9 | 6136 | 41.2 | 16931 | 40.8 |
| Other postgraduate | 1468 | 2.6 | 359 | 2.4 | 1109 | 2.7 |
| Research higher degree | 3909 | 6.9 | 1385 | 9.3 | 2524 | 6.1 |
| **Fee Status** |  |  |  |  |  |  |
| Australian Fee | 6561 | 11.6 | 1534 | 10.3 | 5027 | 12.1 |
| Australian subsidised | 28560 | 50.6 | 7755 | 52.1 | 20805 | 50.1 |
| Fee Exempt | 18 | 0.03 | 5 | 0.03 | 13 | 0.03 |
| Overseas fee | 21253 | 37.7 | 5586 | 37.5 | 15667 | 37.7 |
| **Attendance type** |  |  |  |  |  |  |
| Full-Time | 51872 | 92.0 | 13950 | 93.8 | 37922 | 91.4 |
| Part-Time | 4520 | 8.0 | 930 | 6.3 | 3590 | 8.6 |
| **Study Mode** |  |  |  |  |  |  |
| Standard campus | 55610 | 98.6 | 14726 | 99.0 | 40884 | 98.5 |
| Graduate Online Melbourne | 764 | 1.4 | 149 | 1.0 | 615 | 1.5 |
| Mixed | 18 | 0.03 | 5 | 0.03 | 13 | 0.03 |
| **Commencing status** |  |  |  |  |  |  |
| Commencing | 19214 | 34.1 | 5313 | 35.7 | 13901 | 33.5 |
| Returning | 37178 | 65.9 | 9567 | 64.3 | 27611 | 66.5 |
| **Faculty** |  |  |  |  |  |  |
| Architecture, Building and Planning | 3833 | 6.8 | 883 | 5.9 | 2950 | 7.1 |
| Arts | 9436 | 16.7 | 2535 | 17.0 | 6901 | 16.6 |
| Business and Economics | 9418 | 16.7 | 2122 | 14.3 | 7296 | 17.6 |
| Engineering | 4649 | 8.2 | 1244 | 8.4 | 3405 | 8.2 |
| Fine Arts and Music | 2169 | 3.8 | 570 | 3.8 | 1599 | 3.9 |
| Law | 2224 | 3.9 | 551 | 3.7 | 1673 | 4.0 |
| Medicine, Dentistry and Health Sciences | 8777 | 15.6 | 2592 | 17.4 | 6185 | 14.9 |
| Education | 2940 | 5.2 | 814 | 5.5 | 2126 | 5.1 |
| Science | 11295 | 20.0 | 3033 | 20.4 | 8262 | 19.9 |
| Veterinary & Agricultural Sciences | 1651 | 2.9 | 536 | 3.6 | 1115 | 2.7 |

^1^ Summary statistics derived using central university administrative records. The anonymous survey responses could not be linked with administrative records, only whether students responded or not to the survey. Hence there may be small discrepancies between counts and percentages for these demographic characteristics and those reported by respondents in Table 2. For example, these figures include 17 students who were less than 18 years old according to university records; these students were ineligible and their survey responses were blocked as they did not meet eligibility criteria for the survey. ^2^ Special administrative regions
